# Adherence to PRISMA 2020 statement assessed through the expanded checklist in systematic reviews of interventions: A meta‐epidemiological study

**DOI:** 10.1002/cesm.12074

**Published:** 2024-05-23

**Authors:** Diego Ivaldi, Mariana Burgos, Gisela Oltra, Camila E. Liquitay, Luis Garegnani

**Affiliations:** ^1^ Research Department Instituto Universitario Hospital Italiano de Buenos Aires Buenos Aires Argentina

**Keywords:** methodology, PRISMA, quality, systematic reviews

## Abstract

**Introduction:**

The preferred reporting items for systematic reviews and meta‐analyses (PRISMA) statement was developed to improve the reporting of systematic reviews (SRs) and meta‐analyses. Due to the suboptimal reporting of the 2009 version, an update was performed and published in 2021. Despite having been evaluated in studies published before its publication, its adherence in SRs of interventions published after 2021 remains unclear.

**Objective:**

To assess PRISMA 2020 statement adherence and its uptake in SRs of interventions.

**Methods:**

We conducted a prospective cross‐sectional study searching MEDLINE (PubMed), including a 10% random sample of all SRs involving human interventions published since January 2022 retrieved by our search process. We did not apply any restrictions. We assessed PRISMA 2020 statement uptake and its adherence using its expanded checklist.

**Results:**

We included 222 out of 945 studies. 67 (30.18%) used PRISMA 2020 statement. None adhered completely, with an average adherence of 42.64% (Min–Max: 14.29%–76.19%). Results and Methods sections had low adherence, with 40.57% (Min–Max: 10.45%–98.51%) and 25.55% (Min–Max: 7.46%–55.22%) respectively. The items with the least adherence were: certainty and reporting bias assessment, excluded studies characteristics and search strategy with 7.46% (5/67), 8.96% (6/67), 10.45% (7/67), and 11.94% (8/67) respectively.

**Discussion:**

As in previous studies, our study showed low adherence, mainly to the methods and results sections. However, our study showed a lower adherence, probably due to the use of the expanded checklist to assessed the tools adherence.

**Conclusion:**

We found a low adherence rate to the PRISMA 2020 expanded checklist. Further PRISMA dissemination and targeted audience training are needed to improve SR reporting and quality.

## 1 INTRODUCTION

Evidence‐based medicine (EBM) is defined as the conscientious, explicit, and judicious use of current best evidence in making decisions about the care of patients, by integrating individual clinical expertise, the best available clinical evidence, and the individual patient's preferences [1]. In recent years, the number of biomedical articles has grown exponentially [2], making systematic reviews (SRs) crucial in EBM, synthesizing all updated and available evidence on a specific topic to help healthcare decision‐makers and beyond.[3], This is due to the rigor of SRs methods, which provide a critical  comprehensive and unbiased synthesis from a whole body of literature focusing on reporting data [4, 5, 6, 7, 8]. However, their credibility depends on the quality of the report, which varies from one to another, affecting their interpretation [9].

In 1999 Moher et al. developed The Quality of Reporting of Meta‐analyses (QUOROM) statement, comprising eight sections with 18 itmes [10]. Some studies found a variable adherence rate to QUOROM statement identifying between 3 to 4, and 4 to 8 items with fewer compliance than 67% and 50% respectively [11, 12].

As an update to the QUOROM statement, a guideline for reporting meta‐analysis, The preferred reporting items for systematic reviews and meta‐analyses (PRISMA) statement is a guideline developed to help authors improve the reporting of systematic reviews and meta‐analyses [9].

First published in 2009, there are now several extensions to improve the reporting of essential aspects in these reviews [13, 14, 15, 16, 17, 18, 19, 20, 21], mainly addressing evaluations of interventions [9]. Page et al. found that 57 studies assessed the SRs published between 1989 and 2016 adherence to PRISMA statements, identifying 11 and 6 items with fewer than 67% and 50% of adherence, respectively [22].

Although some studies evaluated different PRISMA extensions, most focused on assessing PRISMA 2009 adherence [22]. This tool was widely adopted, and its reporting was suboptimal, with nine and one items with fewer than 67% and 21% of adherence respectively [22], findings supported by recent studies showing similar results, with seven and nine items with fewer than 67% and 50% of adherence respectively for SRs published between 2011 and 2020 in all rehabilitation journals [23].

Those findings led to an update in 2020 by Page et al. and published in 2021, comprising seven sections with 27 items, some of which include subitems, giving a total of 42 items [13, 22].

There is some evidence about the PRISMA 2020 statement adherence in SRs [24]. This study showed that for the 42 items of the tool, more than 50% of them were adequately reported in less than 80% of the included articles, suggesting further improvements are needed to fulfill PRISMA 2020 statement [24]. However, it focused on reporting the adherence in SRs published between 2009 and 2021 in only one peer‐reviewed journal [24], probably leading to biased conclusions and limiting its generalization.

According to recently exposed evidence, there is an absence of studies assessing PRISMA 2020 statement adherence in SRs of interventions published after its publication date remains to be discovered, which, if poorly reported, could fail to determine their trustworthy and to guide decision making adequately [25].

### 1.1 Objective

To assess PRISMA 2020 statement adherence in SRs of interventions. A secondary objective was to assess PRISMA 2020 statement uptake in those reviews.

## 2 MATERIALS AND METHODS

### 2.1 Study design

We conducted a prospective cross‐sectional study following the guideline for reporting meta‐epidemiological studies, based on adapted PRISMA statement items [26]. We presented partial results from this research at the 2023 Cochrane Colloquium.

### 2.2 Search strategy

We searched the MEDLINE database. We used a design filter to retrieve systematic reviews from validated MacMaster, adapted for Pubmed (Best balance of sensitivity and specificity).

The search strategy was limited to retrieving reviews published from January 1, 2022, to February 23, 2023. No language or place of publication restrictions were applied.

Finally, a 10% random sample was selected from the results obtained to ensure the viability of the study.

Details of the search strategy are available in Table 1.

**Table 1:** Characteristics of included studies.

Variable	Estimate *n* (%)
Year of publication	
‐ 2022	57/67 (85.07)
‐ 2023	10/67 (14.93)
First author country	
‐ Germany	8/67 (11.94)
‐ Spain	7/67 (10.45)
‐ Italy	6/67 (8.96)
‐ China	5/67 (7.46)
‐ United Kingdom	5/67 (7.46)
‐ Australia	4/67 (5.97)
‐ Brazil	4/67 (5.97)
‐ USA	4/67 (5.97)
‐ Others	24/67 (35.82)
Population	
‐ ≥18 years	49/67 (73.13)
‐ <18 years	6/67 (8.96)
‐ Mixed population	9/67 (13.43)
‐ Not reported	3/67 (4.48)
Topic	
‐ Oncologic disorders	9/67 (13.43)
‐ Musculoskeletal disorders	8/67 (11.94)
‐ Orthopedics	7/67 (10.45)
‐ Metabolic disorders	5/67 (7.46)
‐ Respiratory disorders	5/67 (7.46)
‐ Neurologic disorders	4/67 (5.97)
‐ Endocrinologic disorders	3/67 (4.48)
‐ Ophtalmic disorders	3/67 (4.48)
‐ Others	23/67 (34.33)
Intervention	
‐ Physical therapy	21/67 (31.34)
‐ Surgery	17/67 (25.37)
‐ Pharmacological	16/67 (23.88)
‐ Psychological	2/67 (2.99)
‐ Nutritional	1/67 (1.49)
‐ Others	10/67 (14.93)

### 2.3 Eligibility criteria and study selection

We included completed noncochrane (SRs) involving human interventions published since January 2022 reporting using PRISMA statement guidelines. We excluded Cochrane SRs firstly because of their more complete reporting due to their high methodological standards and the rigorous editorial process they have to fulfill [27], and second, because those reviews used to follow the Methodological Expectations of Cochrane Intervention Reviews (MECIR) recommendations, which cover both conducting and reporting review protocols, new reviews and updates of reviews of interventions [28]. We did not apply restrictions based on language or topic.

One reviewer (out of Diego Ivaldi, Gisela Oltra, and Mariana Burgos) screened titles, abstracts, and full texts using Covidence [29], and extracted data from included studies using a specific extraction tool developed for this research with Google Sheets [30]. These processes underwent validation by a second author (Luis Garegnani). We did not contact the study authors.

### 2.4 Data extraction

We assessed the uptake of PRISMA 2020 among all reviews reporting using any PRISMA statement, considering its uptake whenever the authors cited it in Section 2. We assessed PRISMA 2020 statement adherence in those SRs reporting using it by using the PRISMA 2020 expanded checklist[31]. Items were considered adherent if authors reported all the essential elements for each one. In case authors did not report they could not correctly develop any of the items due to their non‐applicability or if they did not prespecify it in a protocol, the item was considered non‐adherent.

Moreover, we used the PRISMA 2020 explanation and elaboration guidance for assisting the adherence decisions when needed (e.g., when one reviewer considered that the mandatory items description of the study to be evaluated was confusing) [31].

For included reviews, we extracted information about the date, country, topic of the published SRs, type of intervention and population. We checked each item of the PRISMA 2020 statement for each included review. Finally, we analyzed the absolute and relative number of adherence to each of the items by each of the SRs and their supplementary material and the general adherence to the PRISMA 2020 statement, considering correct adherence as long as all the mandatory elements of each item are met.

### 2.5 Statistical analysis

We reported continuous variables as means and standard deviations or medians and interquartile ranges according to the distribution, analyzed by visual inspection of histograms, standardized normal probability plots and the Shapiro–Wilk test. We reported categorical variables as absolute numbers and proportions. We used STATA 16.0 software for the statistical analysis (StataCorp LLC) [32].

## 3 RESULTS

**Figure 1:**
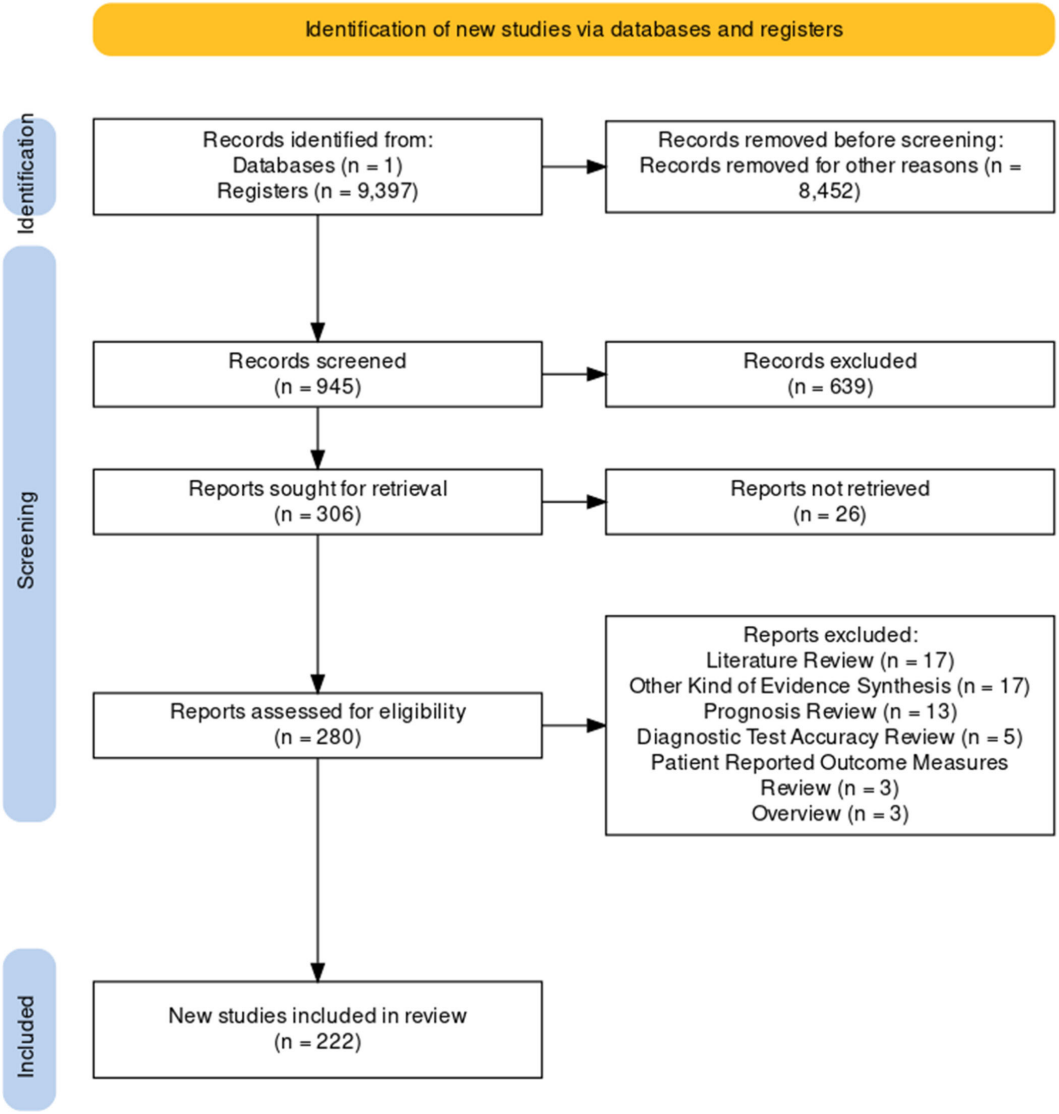
Study flow diagram.

The search retrieved 9397 results. For feasibility, we randomly extracted a sample of a 10% (*n* = 945) for eligibility assessment. After title and abstract screening, we assessed 280 full texts and finally included 222 studies. We excluded 58 studies and described their characteristics in Table 1. A full description of the screening process is shown in Figure 1 [33].

Most SRs used PRISMA 2009 statement to guide their reporting 86 (38.74%). Only 67 (30.18%) studies reported using the PRISMA 2020 statement, including six studies reporting using both PRISMA 2020 and 2009 statements. Besides that, 75 (33.78%) SRs did not report the PRISMA statement version used.

Of the 67 reviews using PRISMA 2020 statement, 57 (85.07%) were published in 2022. Most reviews were published in Europe, followed by Asia and America, Oceania and finally Africa, with 40 (59.70%), 11 (16.42%), 4 (5.97%) and 1 (1.49%) reviews, respectively. A detailed description of the characteristics of the SRs is shown in Table 1.

Considering all items from each SRs reported using the PRISMA 2020 Statement, 1200/2814 items were adequately addressed. Therefore, none of the SRs fully adhered to the PRISMA 2020 statement. On average, SRs adhered 42.64% (Min–Max: 14.29%–76.19%) to the PRISMA 2020 statement. The maximum adherence was found in two SRs, one assessing herbal medicine and physiotherapy interventions for respiratory and musculoskeletal disorders and the other) assessing pharmacological interventions for internal medicine disorders.

Regarding the adherence to different PRISMA 2020 sections, SR adhered the most to the title section items, with 85.57% adherence, followed by the introduction, the discussion, and the other information sections with 85.07% (Min–Max: 73.13%–97.01%), 79.10% (Min–Max: 46.27%–97.01%), and 56.22% (Min–Max: 1.49%–94.03%) respectively. Meanwhile, Sections 2 and 3 adhered in 40.57% (Min–Max: 10.45%–98.51%) and 25.55% (Min–Max: 7.46%–55.22%) respectively. And finally, the abstract one did not adhere at all 0.00%.

About the items, SRs adhered the most to the study characteristics' items with 98.51% (66/67), followed by the rationale, interpretation of the results, and competing interests with 97.01% (65/67), 97.01% (65/67), and 94.03% (63/67) respectively. Additionally, SRs adhered more than 80% to the following four items: the limitations of evidence with 89.55% (69/67), the title and the support with 86.57% (58/67), and the implications with 83.58% (56/67).

**Figure 2:**
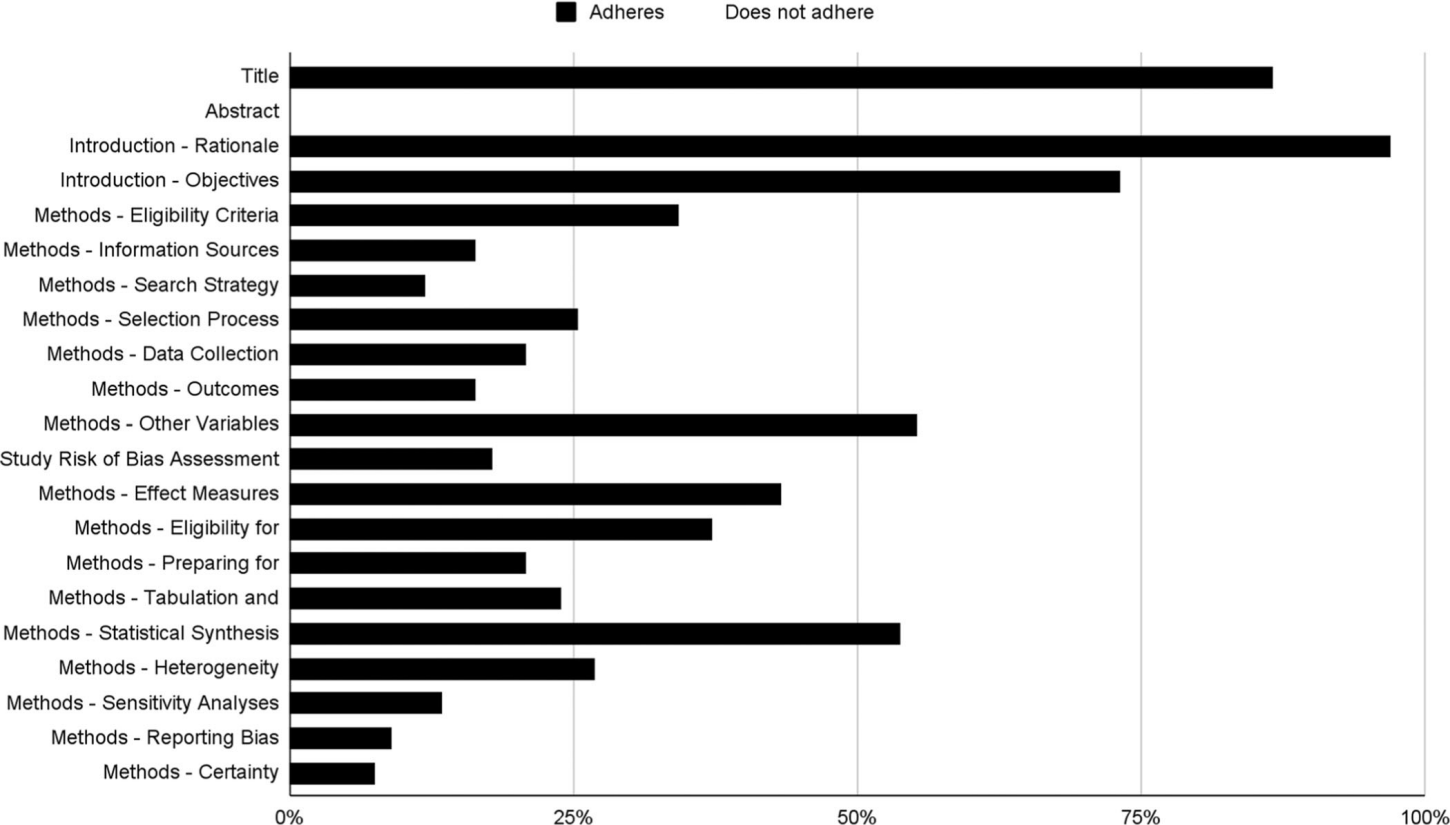
Adherence rate to PRISMA 2020 statement Title, Abstract, Introduction and Methods sections.

**Figure 3:**
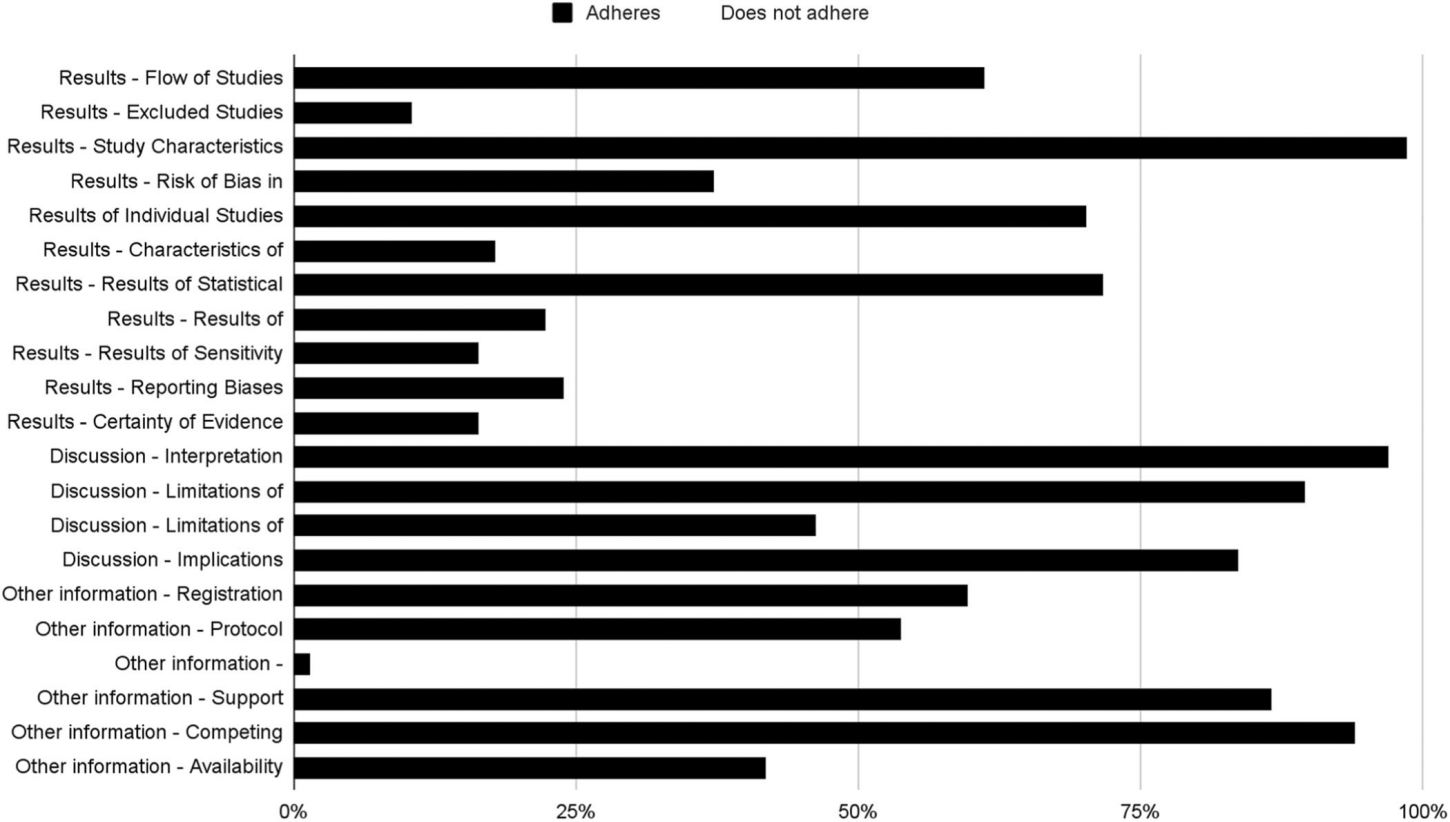
Adherence rate to PRISMA 2020 statement Results, Discussion and Other information sections.

The most problematic items were the amendments with 1.49% (1/67) of adherence, followed by the certainty assessment with 7.46% (5/67), the reporting bias assessment with 8.96% (6/67), the excluded studies characteristics with 10.45% (7/67), and the search strategy with 11.94% (8/67). The remaining characteristics are shown in Figures 2 and 3.

## 4 DISCUSSION

The main finding of our study is that the overall PRISMA 2020 statement adherence in non‐Cochrane SRs involving human interventions is low, mainly due to issues with the methods and results sections, and found to be lower than the ones reported by Page et al. and Park et al., [22, 24] however those previous studies are not completely comparable due to different focus. In our cohort, SRs adhered more than 80% to only eight items, unlike what was reported by Page et al. and Park et al., [22, 24] with 12 and 18 items reporting an adherence greater than 80% respectively. One possible explanation for such compliance differences could be that Page et al., [22] focused in the PRISMA 2009 statement adherence, while our study focused in the PRISMA 2020 statement, a recently developed and published tool. Furthermore, although Park et al. assessed the PRISMA 2020 statement adherence including studies published before its publication, they did not use the expanded checklist [22, 24], which could have increased their adherence rate due to not taking into account compliance to mandatory elements.

In our study, included SRs did not adhere at all to the abstract section's item, differing from the findings from Page et al. and Park et al. [22, 24] This could be explained since, on one hand to fullfil the PRISMA 2009 statement abstract item, it is enough to simply provide a structured abstract instead of fulfilling the whole PRISMA for abstracts extension. On the other hand, Park et al. focused their assessment on each abstract item separately [24]. Likewise, despite being a critical section, adequate adherence of PRISMA elements for abstracts varies widely from approximately 0% to 98% depending on the literature [34, 35, 36, 37, 38].

As reported by Page et al. and Park et al., [22, 24] our study showed a low adherence rate to the methods and results sections' items. This may be related to the complexity of those section items and journals' editorial conditions, such as the word limits, that may lead author to shorten part of the method section information and some parts of the results section such as the description of the excluded studies, to focus on the results of their research. However, our study showed a lower adherence, which could be attributed to the strictest criterion we use when evaluating the adherence rate using the expanded checklist.

Our study is not free from limitations. First, our study lacks of a prospectively registered protocol. Second, the SR's authors may have been unaware of the new version of the PRISMA statement since its publication was in February 2021, limiting its uptake and proper implementation or adherence. Third, we decided to randomly select a 10% sample from the final search results, seeing that the decision to include all noncochrane systematic reviews involving human interventions without topic or journal restriction would yield an unmanageable number of results. Finally, we did not implement an independent study selection and data extraction process due to feasibility issues, with only one out of all the study authors conducting these stages. To secure quality, we implemented a second reviewer validation process.

Further studies are needed focusing on dissemination and training on PRISMA 2020 statement to improve the SRs reporting, thus increasing their credibility. Researchers should consider allocating additional resources to the final reports' elaboration and building capacity in writing and editing within their teams. Finally, new prospectively registered studies evaluating PRISMA 2020 statement adherence are needed, including different approaches, assessing its adherence in a general and specific way, and analyzing factors that may be associated with its adherence or non‐adherence.

## 5 CONCLUSION

The PRISMA 2020 statement uptake rate is low and was lower than the PRISMA 2009 statement. The overall PRISMA 2020 statement adherence was also low. As reported in previous studies assessing both PRISMA 2009 and PRISMA 2020 statement, the present study found that the methods and results items fail to adhere completely to the PRISMA 2020 expanded checklist. To improve the quality of reporting SRs, authors should be familiar with the tool and know how to report its different sections properly. Further dissemination and training, by using PRISMA 2020 explanation and elaboration are needed to improve their reporting.

## AUTHORS CONTRIBUTIONS

**Diego Ivaldi**: Study conception and design; data collection; analysis and interpretation of results; draft manuscript preparation. **Mariana Burgos**: Data collection; draft manuscript preparation. **Gisela Oltra**: Data collection; analysis and interpretation of results. **Camila E. Liquitay**: Study conception and design; draft manuscript preparation. **Luis Garegnani**: Study conception and design; analysis and interpretation of results; draft manuscript preparation. All authors reviewed the results and approved the final version of the manuscript.

## CONFLICT OF INTEREST STATEMENT

The authors declare no conflict of interest.

## Data Availability

The data that support the findings of this study are available on request from the corresponding author.
